# Serological insights of Crimean-Congo haemorrhagic fever virus in podolic cattle bred in Italy

**DOI:** 10.3389/fcimb.2026.1810497

**Published:** 2026-04-21

**Authors:** Donato Antonio Raele, Kerstin Fischer, Julia Holzerland, Caroline Bost, Domenico Scaltrito, Antonio Parisi, Donatella Belluscio, Anna Mattea D’Antuono, Lara Caprarella, Antonella Narducci, Goffredo Sarti, Domenico Pugliese, Michela Sordillo, Guido Di Donato, Maria Assunta Cafiero, Nicola Cavaliere

**Affiliations:** 1 Istituto Zooprofilattico Sperimentale della Puglia e della Basilicata, Foggia, Italy; 2 Friedrich-Loeffler-Institute, Institute of Novel and Emerging Infectious Diseases, Federal Research Institute for Animal Health, Greifswald, Germany; 3 Azienda Sanitaria Locale, SIAV-A, Foggia, Italy; 4 Istituto Zooprofilattico Sperimentale dell’Abruzzo e del Molise “G. Caporale”, Teramo, Italy

**Keywords:** cattle, CCHFV, Italy, serologic analysis, virus neutralization test (VTN)

## Abstract

We evaluated the presence of antibodies against *Orthonairovirus haemorrhagiae*, formerly Crimean-Congo haemorrhagic fever virus (CCHFV) in the serum of podolic cattle from southern Italy. A subset of 20 sera (18 positive and 2 negative) from the initial 670 bovine samples screened by commercial ELISA kit were further evaluated by indirect ELISA and virus neutralization tests. Across the three serological assays, antibodies against CCHFV were detected in a proportion of samples, though the results were not fully concordant and varied according to the assay applied. These findings highlight the need for multiple tests to more accurately assess CCHFV circulation in free-range cattle, particularly in non-endemic areas, and emphasize the importance of careful interpretation when relying on serology alone.

## Introduction

Crimean-Congo haemorrhagic fever (CCHF) is a severe tick-borne zoonosis characterized by high case-fatality rates, reaching up to 40% in some outbreaks, and a vast geographical footprint spanning Africa, Asia, the Middle East, and South-Eastern Europe (Bernard et al., 2022; Eslava et al., 2024). The causative agent is a member of the Orthonairovirus genus primarily transmitted by ticks of the *Hyalomma* genus, which serve as both biological vectors and reservoirs. These ticks can infect a broad spectrum of wild and domestic animals; while these hosts typically remain asymptomatic, they develop a transient viremia, acting as crucial amplifying hosts for the virus (Bente et al., 2013; Celina et al., 2025). Domestic ruminants, particularly cattle, sheep, and goats, play a crucial role in the CCHFV life cycle by facilitating virus transmission to immature and adult ticks during blood feeding (Bernard et al., 2025; Maze et al., 2025). Furthermore, these animals serve as essential sentinels for public health surveillance, as their seroprevalence often precedes the emergence of human cases. In recent years, the geographical range of *Hyalomma* ticks has expanded toward higher latitudes, driven by climate change and the migration of infested birds, raising concerns about the potential establishment of the virus in previously unaffected regions (Romanek et al., 2025). Although Italy is currently classified as a non-endemic area with no reported human cases to date, the epidemiological landscape is evolving. Recent findings of CCHFV RNA in *Hyalomma rufipes* ticks and serological evidence in livestock across southern regions suggest that the virus may already be circulating in cryptic cycles (Mancuso et al., 2019; Fanelli et al., 2022). Given these premises, enhancing serological surveillance in domestic animals is imperative to assess the actual risk of CCHFV introduction and to implement early-warning systems within a ‘One Health’ framework.

## The study

This study was based on 670 bovine serum samples collected between 2024 and 2025 from 59 farms in the Gargano National Park (Apulia region). All farms followed a free-range husbandry system for Podolic cattle in areas where *Hyalomma* ticks are present. Using the ID Screen^®^ CCHF Double Antigen Multi-species (ET1), 105 samples tested positive, resulting in an individual seroprevalence of 15.7% (105/670) and a herd-level prevalence of 39% (23/59). The sample size was determined using Epi Info™ v7.2 (CDC). Assuming an infinite population, an expected CCHF seroprevalence of 25% (based on 2023 data), a 95% confidence level, and a 5% margin of error, the initial sample of 289 subjects was adjusted by a design effect (DEFF) of 1.27 to account for intra-herd clustering, requiring a minimum of 367 cattle. A subset of 20 serum samples was selected to represent the geographic diversity of the study area, including 10 different farms. To rigorously assess the concordance between tests, the selection included a range of antibody titres, from strong positives to borderline cases. Two negative controls were specifically chosen from a farm with confirmed positive cases in neighbouring areas, to strictly evaluate the test’s analytical specificity under real environmental exposure conditions (i.e., presence of ticks). In our study, the serological diagnostic accuracy for CCHFV was assessed using 18 positive and 2 negative samples collected from 10 different farms in the study area and previously screened by ET1 using manufactory instructions. In addition, to test for anti-CCHFV N (nucleocapsid protein) IgG using another test principle, cattle serum samples were tested using an indirect in-house ELISA based on the CCHFV (Kosovo Hoti) nucleoprotein (ET2), recombinantly expressed and purified as described in Schuster et al., 2016 (Schuster et al., 2016). For that, 96-well Greiner F microlon plates were coated with 200 ng/well of recombinant CCHFV N-his, diluted in 0.01 M PBS (pH 7.4) with 0.5% bovine serum albumin (BSA; Roth Germany), and incubated overnight at 4 °C. Plates were washed once with 250 μL washing buffer (PBS with 0.1% Tween20, Sigma-Aldrich; PBST) and blocked with blocking buffer for 1 h at 37 °C. Each bovine serum sample was diluted 1:80 in Dilution Buffer (IDvet) and added in duplicate to control- and antigen-containing wells (100 μL/well). After incubation for 1 h at 37 °C, plates were washed three times with PBST before goat-anti-bovine IgG horseradish peroxidase (HRP) conjugate (JacksonImmunoR) was added in a dilution of 1:5,000 in Dilution Buffer and incubated for another 1 h at 37 °C. After three washes with PBST, 3,3′,5,5′-tetramethylbenzidine (TMB) peroxidase substrate (Bio-Rad, Munich) was added. The reaction was stopped after 10 min at RT with equal amounts of 1 M sulfuric acid. Absorbance was measured at 450 nm against 620 nm in a Tecan Infinite200Pro ELISA Reader (Tecan GmbH). Corrected OD value (corrOD 450nm) was obtained by subtracting the average OD value of the reaction without antigen (control wells) from the reaction with antigen. Sera were considered reactive against CCHFV N, if the corrOD 450nm exceeded 19% of the corrOD450nm of the positive control, as described in Mertens et al., 2015 (Mertens et al., 2015). To test for neutralizing activity of cattle serum, samples were incubated at 56 °C for 30 min to inactivate complement. Each serum sample was tested in triplicate, serially diluted from 1:4 to 1:32, and added to the wells of an empty 96-well plate. Serial dilutions were then mixed with approximately 150 pfu of CCHFV [Kosovo Hoti] per well and incubated for 1 h at 37 °C. Serum control wells, to which no virus was added, were included to assess potential cytopathic effects (CPE). After 1 h of incubation at 37 °C, 100 µl per well of the serum-virus mix was added to SW13 cells cultured in 96-well plates. Following 7 days of incubation at 37 °C, wells were examined for signs of CPE. Neutralizing activity was assumed in wells where CPE was completely neutralized. The neutralization titre of a serum was calculated as geometric mean titre (GMT) of three replicates that exhibited neutralizing activity. To account for background effects, only serum samples with a neutralizing titre greater than 1:8 were considered positive, as sera from CCHFV-naïve bovine reference animal shave shown to exhibit apparent neutralization up to this dilution. Individual and herd−level prevalence were calculated as proportions with corresponding 95% confidence intervals (CIs) using the Wilson method. Continuous variables, including ELISA OD values, were summarized using means and 95% CIs. In order to evaluate the agreement between the three serological assays employed in this study, we performed a non−parametric Spearman’s rank correlation analysis (Spearman, 1904). This method was selected because it does not assume normal distribution of the variables and is robust to outliers and non−linear relationships conditions particularly relevant given the skewed distribution of VNT titres and the heterogeneous dynamic range of ELISA outputs. Statistical analyses were performed using R software (version 4.3.2; R Foundation for Statistical Computing, Vienna, Austria). Because the analysis focused on effect size (ρ) rather than hypothesis testing, no significance threshold was applied. Mean ET1 and ET2 OD values were 1.03 (95% CI: 0.76–1.29) and 0.69 (95% CI: 0.34–1.04), respectively. Among the 18 sera that initially tested reactive in the ID Screen^®^ ELISA, 13 exceeded the cut-off in the in-house indirect ELISA. Of these 18 reactive samples, 16 were further evaluated using VNT. Neutralizing activity against CCHFV (genotype V) was detected in three of the 16 bovine serum samples, with titres of 1:10 (n = 2) and 1:16 (n = 1) (**Table 1**). The analysis revealed a moderate monotonic association between the ET2 and the VNT (ρ = 0.57), while weaker correlations were observed between the ET1 and both the ET2 (ρ = 0.31) and the VNT (ρ = 0.37). The two serum samples that previously tested negative in the ET1 were confirmed negative by both the ET2 and the virus neutralization test (VNT).

**Table 1:** Results of the three serological assays used to detect exposure to Crimean−Congo hemorrhagic fever virus (CCHFV) in cattle.

IZSPB ID	ELISA IDVet (ET1)	Ind ELISA CCHFV_N (ET2)	VNT CCHFV
	% competition	OD	Dilution titre
92-330840	**50%**	-0,00085	Negative
88-247703	**108%**	0,00170	Negative
94-314160	**172%**	0,00030	Negative
78-247702	**97%**	-0,00245	Negative
2707	**52%**	**0,48910**	Negative
3058-2	**71%**	**0,50420**	Negative
2613-30	**152%**	**2,20300**	Negative
2613-31	**144%**	**2,25025**	**1:16**
2613-29	**115%**	**1,46305**	Negative
2613-32	**121%**	**1,84605**	**1:10**
2399-19	**185%**	**1,12975**	**1:10**
2524-8	**98%**	**0,25935**	Negative
2525-19	**99%**	**0,69645**	not performed^α^
330-CAM	**192%**	0,00220	Negative
3258-31	**46%**	**0,42305**	Negative
3258-13	**64%**	**1,07385**	not performed^α^
3258-2	**144%**	**0,94530**	Negative
3258-27	**141%**	**0,33505**	Negative
1389-15	0%	0,06600	Negative
1389-16	0%	0,05240	Negative

For each serum sample, results obtained with the commercial ID Screen^®^ CCHF Double Antigen Multi−species ELISA (ET1), the in−house indirect ELISA targeting the CCHFV nucleoprotein (ET2), and the virus neutralization test (VNT) are reported. ET1 results are expressed as percentage competition, with values >30% considered positive. ET2 results are expressed as optical density (OD), with samples exceeding 19% of the positive control classified as reactive. VNT titres represent the highest serum dilution fully inhibiting cytopathic effect; titres >1:8 were interpreted as evidence of neutralizing antibodies. Bold values indicate positive results according to the respective assay cut−offs. Samples marked with α were not tested by VNT due to insufficient serum volume.

## Conclusions

**Figure 1:**
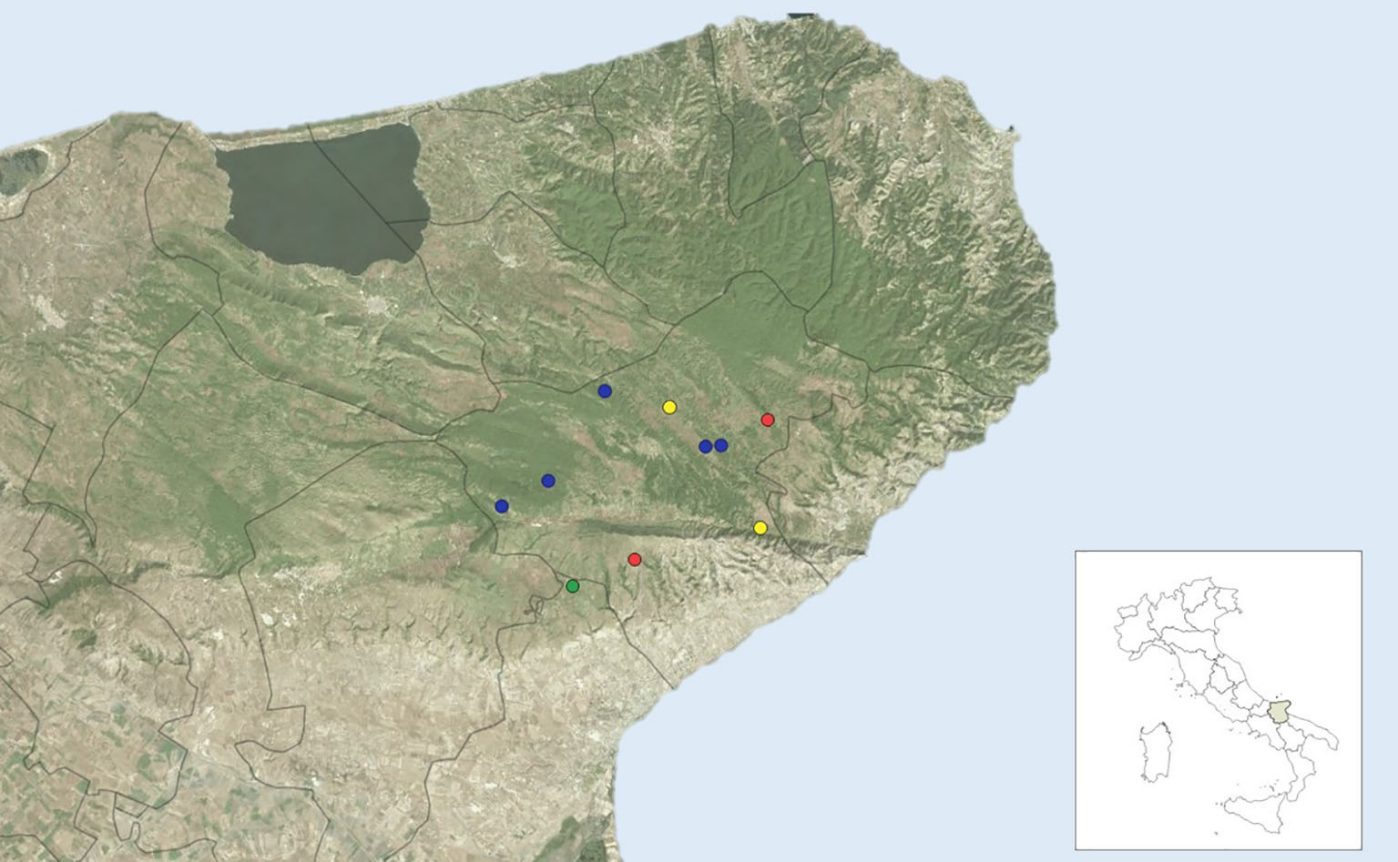
This spatial representation highlights the heterogeneity of serological reactivity within the study area and supports the interpretation of localized exposure patterns to CCHFV or antigenically related Orthonairoviruses. Farms are represented by coloured markers indicating the highest level of diagnostic evidence obtained among the three assays used in this study. Yellow markers: farms with animals testing positive only in the commercial ID Screen^®^ ELISA (ET1); Blue markers: farms with animals positive in both ET1 and the in−house indirect ELISA targeting the CCHFV nucleoprotein (ET2); Red markers: farms with animals positive in all three assays, including the virus neutralization test (VNT), indicating the presence of neutralizing antibodies; Green marker: farm in which all tested animals were seronegative across all assays.

From a total of 59 farms investigated, 10 distinct farms were selected for an in-depth serological assessment. Among these, 9 out of 10 herds of podolic cattle showed serological reactivity to at least one CCHFV screening test (**Figure 1**). Although all three samples exceeded the established VNT threshold of 1:8 for bovine sera, the observed neutralizing titres (1:10 and 1:16) are considered low. This weak neutralizing activity may reflect prior exposure to a heterologous CCHFV strain that cross-reacts only minimally with the genotype V virus used in this study, which aligns with current findings indicating that Africa 3 is the only CCHFV genotype recorded in Italy (Romanek et al., 2025). Alternatively, it may suggest exposure to a closely related Orthonairovirus, such as Aigai virus (formerly Crimean-Congo haemorrhagic fever virus genotype VI), which could elicit weakly cross-neutralizing antibodies (Papa et al., 2022). Another possible explanation is waning antibody levels, as the kinetics, durability, and longevity of the antibody response in cattle following CCHFV infection remain poorly understood. Regarding the ET1 results, five sera were considered positive but were not confirmed by either the ET2 or the VNT test. Again, the lack of confirmation could be due to the test having a lower specificity than expected or, alternatively, to a higher analytical sensitivity. Our results indicate that, although all assays contribute valuable information, the ET2 shows the strongest concordance with neutralizing antibody activity. This supports its potential utility as a surrogate marker for functional immunity in epidemiological investigations of CCHFV exposure. Taken together, our data provide serological evidence of contact between putative viral amplifier hosts and CCHFV or a closely related Orthonairovirus in the Gargano area, without indicating the establishment of a sustained enzootic transmission cycle in Italy. It is therefore clear that in non-endemic areas, serological investigations of domestic ruminants must be as detailed as possible to avoid false positive or negative results in animals sentinel. Moreover, molecular evidence, such as detection of the viral genome in ticks, are required to confirm the serological evidences.

## Data Availability

The raw data supporting the conclusions of this article will be made available by the authors, without undue reservation.
